# Supplementary material for: Cloud BioLinux: pre-configured and on-demand bioinformatics computing for the genomics community
Source: BMC Bioinformatics. 2012 Mar 19;13:42. doi: 10.1186/1471-2105-13-42 (PMC3372431; doi:10.1186/1471-2105-13-42)
Supplement: Additional file 1 — Supplementary 1 Cloud BioLinux software documentation in the form of a mini, self-contained website. Users need to download and uncompress the .zip file, and open through a web browser the "index.html" file available on the main directory. (ZIP 1823 kb). [file 1471-2105-13-42-S1.ZIP › Cloud-BioLinux-Package-Documentation/docs/assembly-conversion-tools.html]

Bio-Linux Software Documentation Pages

Back to search form

## assembly-conversion-tools

|  |  |
| --- | --- |
| Name | assembly-conversion-tools |
| Description | The bio-linux-assembly-conversion-tools package contains programs that will convert sequence assemblies so they can be loaded into software such as Staden's Gap4 or the Consed assembly viewer.  Information on how to use the scripts provided in this package is available through the documents listed in the Remote Documents section of this page.  The scripts included in this package currently are:   - roche454ace2gap.sh - a script allowing the conversion of 454 Newbler assemblies into a format for loading into Gap4. - caf2gap - converts assemblies in Common Assembly Format into Gap4 format.   We hope to extend the tools in this package in the future. If you know of useful assembly conversion tools that you think should be included, please contact us through the NEBC helpdesk. |
| Homepage |  |
| Remote Documentation | http://envgen.nox.ac.uk/envgen/software/archives/000581.html#staden      http://genome.imb-jena.de/software/roche454ace2caf/ |
